# Random-Pattern Skin Paddle on a Free Latissimus Dorsi Flap as an Intraoperative Backup for Distal Lower-Limb Reconstruction: A Case Report

**DOI:** 10.3390/clinpract16060102

**Published:** 2026-05-28

**Authors:** Ivan Budimir, Borna Vojvodić, Rado Žic, Zlatko Vlajčić, Domagoj Eljuga, Božo Gorjanc, Željka Roje, Hrvoje Tucaković, Željka Godeč, Marko Barić, Josip Jaman, Rhea Marie Mužar, Krešimir Martić

**Affiliations:** 1 Department of Plastic, Reconstructive and Aesthetic Surgery, University Hospital Dubrava, Avenija Gojka Šuška 6, 10000 Zagreb, Croatia; 2 School of Medicine, University of Zagreb, Šalata 2, 10000 Zagreb, Croatia; 3 Faculty of Medicine, Josip Juraj Strossmayer University of Osijek, Josipa Huttlera 4, 31000 Osijek, Croatia; 4 Faculty of Dental Medicine and Health, Josip Juraj Strossmayer University of Osijek, Crkvena ul. 21, 31000 Osijek, Croatia; 5 Department of Hand Surgery, Clinic for Traumatology, Sestre Milosrdnice University Hospital Center, Draškovićeva 19, 10000 Zagreb, Croatia; 6 Nursing Department, Bjelovar University of Applied Sciences, Trg Eugena Kvaternika 4, 43000 Bjelovar, Croatia; 7 Nursing and Physiotherapy Department, University North, Jurja Križanića 31b, 42000 Varaždin, Croatia

**Keywords:** reconstructive surgery, latissimus dorsi free flap, random-pattern skin paddle, complex wound coverage, soft tissue defect

## Abstract

**Background:** The latissimus dorsi free flap is a workhorse for extensive lower-extremity soft tissue defects. Conventionally, the skin paddle is designed according to the anticipated defect and left in place on the muscle as a single composite unit. This report describes an alternative approach in which the skin paddle is secondarily mobilized through subcutaneous undermining and rotated as a separate propeller-type local extension flap on random-pattern vascularization, without a specifically identified perforator—a technique that has not been previously reported. **Case Presentation:** A 38-year-old male with a high-energy distal lower-extremity defect exposing bone, Achilles tendon, and hardware underwent free latissimus dorsi reconstruction with an empirically designed skin paddle over the constant perforator zone. The skin paddle was subsequently mobilized and rotated as a separate propeller-type extension flap to cover the Achilles region, with additional areas managed using split-thickness skin graft and a reverse soleus flap. **Results:** The latissimus dorsi flap and skin paddle remained viable, providing stable coverage of the defect. The additional reverse soleus flap achieved durable medial coverage, and the limb was ultimately preserved with satisfactory soft-tissue stability. **Conclusion:** A random-pattern latissimus dorsi skin paddle designed within the anatomically constant perforator zone can provide a feasible new option offering intraoperative flexibility in complex lower-extremity trauma when perforator mapping is impractical.

## 1. Introduction

The latissimus dorsi muscle was first described by Tansini in 1896 for closure of mastectomy defects and subsequently introduced for lower extremity microvascular reconstruction by Watson and colleagues in 1979 [1]. The latissimus dorsi is the largest muscle in the body, yet remains relatively thin (<1 cm thick), and is classified as a Type V muscle according to the Mathes and Nahai vascular classification system, being supplied principally by the thoracodorsal artery as the dominant pedicle and secondary segmental pedicles from the posterior intercostal and lumbar arteries [1,2]. The thoracodorsal artery is a terminal branch of the subscapular artery, entering the deep surface of the latissimus dorsi muscle at the neurovascular hilus approximately 10 cm inferior to the axillary artery, and supplies the superolateral half of the latissimus dorsi muscle to its insertion, with additional branches to the serratus anterior and teres major muscles and often a large direct cutaneous branch that supplies skin over the anterosuperior border of the muscle [1,3].

The latissimus dorsi free flap remains a workhorse option for extensive soft tissue defects of the lower extremity, particularly in scenarios where local and pedicled flaps are exhausted due to zone-of-injury extent or defect size [1]. It offers vessels with a long reliable pedicle, rapid and safe dissection due to predictable anatomical landmarks, the ability to be transferred as muscle, musculocutaneous, or composite flap, and the versatility to be tailored to almost any size and shape to match the recipient defect [1]. Recent cadaveric studies have demonstrated that within a defined constant perforator zone located 9.5 to 15.4 cm below the posterior axillary fold and within 4.3 cm of the lateral border, at least one musculocutaneous perforator ≥ 0.5 mm in diameter is present in 100% of specimens. This anatomical consistency supports empirical skin paddle design over this region; however, in vivo factors such as high-energy trauma, burns, scarring, or previous surgery may disrupt local vascular anatomy, so the “constant perforator zone” concept cannot be assumed to ensure safe design in all cases and should be applied with caution in clinical practice [4]. Furthermore, the latissimus dorsi exhibits three distinct intramuscular vascular territories, with the first territory supplied by the thoracodorsal artery and lateral intercostal perforators representing the primary perfusion zone, and anatomical studies demonstrating that a second territory can be perfused distally when the first territory is adequately supplied [5,6]. In complex high-energy lower-extremity injuries where a single flap is insufficient, chimeric free flaps have been described as a means to extend reconstructive reach, but at the cost of longer operative time and increased complexity [1,7]. An approach that maximizes the versatility of a single free latissimus dorsi flap by using its skin paddle as a secondary local extension may therefore offer a simpler alternative while preserving reconstructive flexibility [8]. This case report describes a novel application of the free latissimus dorsi flap in which the skin paddle, designed empirically over the constant perforator zone without preoperative perforator imaging, was secondarily mobilized through subcutaneous undermining and rotated 150 degrees as an independent propeller-type local extension flap to extend coverage beyond the primary muscle footprint, while acknowledging that there is inherent ischemia risk and that flap safety depended on intraoperative perfusion assessment.

## 2. Case Report

A 38-year-old male was admitted to the emergency department following a motorcycle collision with another vehicle. The patient was hemodynamically stable at presentation (BP 133/77 mmHg, HR 130/min, SpO_2_ 99%) with a Glasgow Coma Scale of 15. The patient’s past medical history was significant for hypertension, and preoperative assessment revealed normal neurological status. He had no history of diabetes or tobacco use. The primary injury involved a severe open fracture of the left lower extremity with an anteromedial wound to the left femur measuring 6 × 4 cm and sure signs of fracture to both upper and lower leg. Immediate temporizing measures included tetanus prophylaxis (Ana-Te + HIG intramuscularly), application of a Kramer splint for immobilization, and urinary catheter placement.

Computed tomographic angiography showed distal opacification only of the posterior tibial artery on the injured left leg, with multifragmentary fractures of the left femoral diaphysis, distal tibia and fibula, anterior tibial malleolus, and bilateral calcaneal fractures. No other acute injuries were identified.

**Figure 1:**
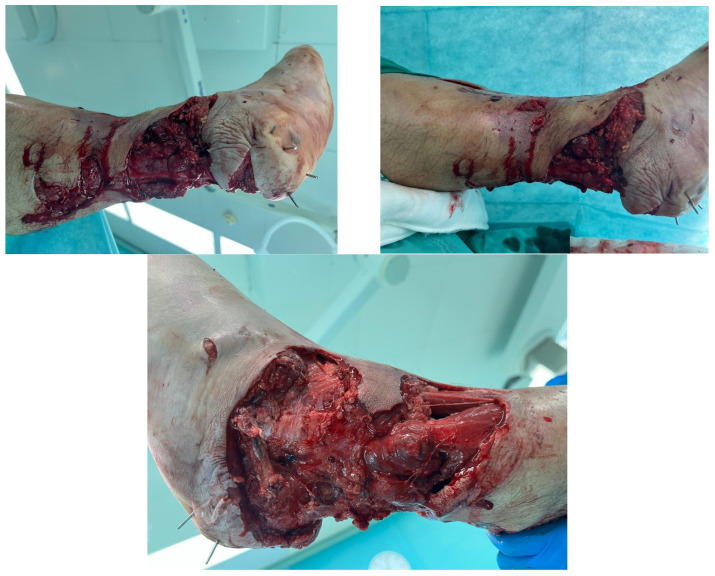
Lateral, posterior and medial aspect of the defect.

The patient underwent staged surgical reconstruction. Firstly, external fixation of the left upper and lower leg was performed. Secondly, definitive orthopaedic reconstruction was undertaken with diagnoses of open comminute fracture of left femoral diaphysis (Gustilo–Anderson Grade II), open comminute fracture of left lower leg (Gustilo–Anderson Grade IIIB), and open fracture of left calcaneal tuber (Gustilo–Anderson Grade IIIB). Surgical procedures included necrectomy of devitalized muscle and soft tissue, intramedullary osteosynthesis of the left femur, intramedullary osteosynthesis of the left tibia, intramedullary osteosynthesis of the left fibula with Kirschner wire, osteosynthesis of the calcaneal tuber with transfixation of medial and lateral calcaneal processes. Calcaneal region also had the avulsion of soft tissues inferiorly involving plantar fascia along with inferior calcaneal cortex and that was managed by repositioning these tissues and fixating it with three Kirschner wires through all mentioned layers. The patient was admitted directly to the surgical intensive care unit postoperatively. Thirdly, because the extent of necrosis and zone of injury remained incompletely demarcated at the time of the first procedures, a second necrectomy was performed to ensure complete removal of nonviable tissue, and negative-pressure wound therapy was applied to the defect pending definitive soft tissue reconstruction. Soft tissue defect was circumferential in the distal lower leg, extending from the lateral malleolus, with exposed Achilles tendon with preserved paratenon and exposed metalwork at the medial malleolus. On both the medial and lateral sides the soft tissue defect extended more proximally, up to the mid–lower leg, with the superficial peroneal nerve exposed laterally. The defect measured approximately 25 × 20 cm in its primary dimensions with exposure of the anterior tibial bone and internal fixation hardware (Figure 1).

At the time of injury, our orthoplastic service was still being established, and initial management was led by the trauma team without a plastic surgeon on site. Definitive osteosynthesis had already been performed before plastic surgery involvement, so in the setting of a high-energy multiplanar injury and no intraoperative perfusion assessment (e.g., ICG) [9], we adopted staged, conservative debridements while aiming to preserve limb-salvage options, accepting further demarcation of the zone of injury as likely, and recognizing the need for flap coverage of exposed osteosynthetic material.

Finally, definitive soft tissue coverage was performed with a free latissimus dorsi musculocutaneous flap. The procedure was performed under general anesthesia. The patient was positioned in the right lateral decubitus position to expose the left lower extremity defect while simultaneously allowing access to the left latissimus dorsi donor site. The left anterior tibial artery and vein were identified and carefully dissected under loupe magnification with meticulous attention to preservation of the superficial peroneal nerve.

The latissimus muscle was carefully dissected with respect to anatomical layers and neurovascular structures, including careful identification and sparing of branches to the serratus anterior muscle to preserve serratus function. Dissection proceeded in the subfascial plane from medial to lateral, with meticulous preservation of the thoracodorsal nerve and intramuscular perforators within the constant perforator zone, and hemostasis was secured using bipolar cautery along the deep surface of the muscle. The muscle was detached from its origin on the spinal processes (T6–T12) and iliac crest and from its insertion on the humerus, allowing the pedicle to be separated on the thoracodorsal artery and vein.

The thoracodorsal artery and vein were meticulously dissected, with all branches to the serratus anterior muscle carefully identified and preserved through selective ligation or bipolar electrocautery. The pedicle was irrigated with a heparinized saline solution (5000 IU heparin per 100 mL) warmed to 37 °C. The flap was then protected between moist swabs and prepared for transfer. No reliable perforator signal was detected on Doppler assessment due to frictional burns in the medial portion of the flap area from the initial trauma, which obscured normal skin landmarks and rendered perforator identification unreliable.

Due to the mentioned circumstances it was elevated en bloc along with the skin paddle which was positioned more posteriorly than usual with no specific perforator identified. Although positioned over the constant perforator zone, the skin paddle was designed empirically without Doppler confirmation of individual perforators and is therefore classified as random-pattern in accordance with established terminology, a strategy supported by cadaveric and translational studies demonstrating a constant musculocutaneous perforator zone and reliable perfusion of latissimus dorsi skin paddles designed according to these principles [4,5,10]. No intraoperative perfusion assessment device such as indocyanine green angiography was available in our institution at the time of surgery. Flap and skin paddle viability were therefore judged using standard clinical parameters, including capillary refill, skin color and temperature, and bleeding from the edges during elevation and mobilization. The idea was to use it somehow in the injured area because skin on that back area was sparable and comprehensive use of STSG or FTSG was expected.

The intraoperative decision-making followed a stepwise logic: (1) the flap was harvested en bloc with an empirically designed skin paddle positioned over the constant perforator zone, given that Doppler assessment was uninformative; (2) after transposition and partial insetting of the muscle component, the defect was reassessed and the distal skin paddle was identified as suitable for Achilles coverage based on the presence of viable paratenon; (3) stepwise subcutaneous undermining of the skin paddle from the underlying muscle was performed under continuous perfusion monitoring, proceeding incrementally until approximately 80% mobility was achieved; (4) the decision to proceed with full rotation was based on concordant clinical indicators of perfusion, including active bright red bleeding from the distal tip together with satisfactory color, warmth, and capillary refill; and (5) the skin paddle was rotated 150° into the Achilles region and inset without tension.

Anastomosis was performed with the latissimus dorsi thoracodorsal vein to the left anterior tibial vein (terminolateral suturing to preserve distal tibial venous outflow) and the thoracodorsal artery to the left anterior tibial artery (terminoterminal anastomosis). After release of vascular clamps, immediate brisk arterial inflow and venous return were observed, confirming satisfactory microvascular anastomoses without bleeding from the suture lines.

**Figure 2:**
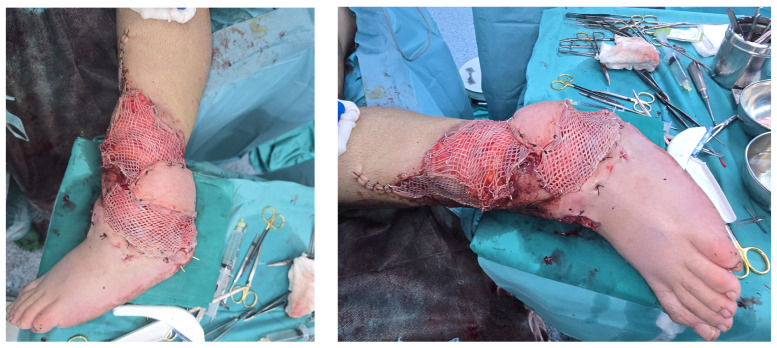
Postoperative appearance following reconstruction.

The latissimus dorsi muscle was inset at the recipient site over the anterior defect with sutures to the medial and lateral margins to secure the muscle coverage over the exposed bone and hardware. The skin paddle was then mobilized from its baseline position and rotated 150 degrees to address a secondary defect involving exposure of the Achilles tendon and posterior heel plantar surface. Exposed soft tissue regions not covered by the latissimus dorsi muscle or the rotated skin paddle were covered with split-thickness skin graft harvested from the anterolateral thigh and meshed at a 1:1.5 ratio (Figure 2).

**Figure 3:**
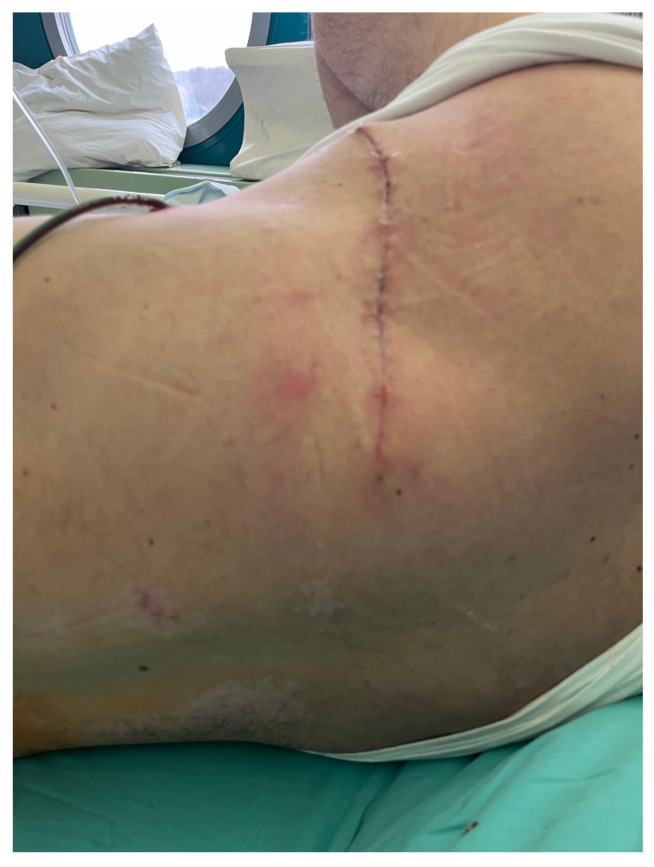
Donor site showing tension-free primary closure.

The donor site was closed in layers to obliterate dead space. Because the latissimus dorsi harvest was limited in extent (respecting the 12th rib boundary and avoiding excessive distal extension) and because the serratus anterior muscle was carefully preserved with intact innervation, the donor site was amenable to primary closure, avoiding the need for split-thickness skin grafting on the back. Two closed suction drains were placed, one in the axilla and one at the lateral chest, to prevent hematoma and seroma accumulation (Figure 3). Drains were removed when output was consistently below approximately 30–40 mL per 24 h, and the donor site was monitored clinically for seroma, wound dehiscence, or shoulder restriction, none of which occurred in this case.

**Figure 4:**
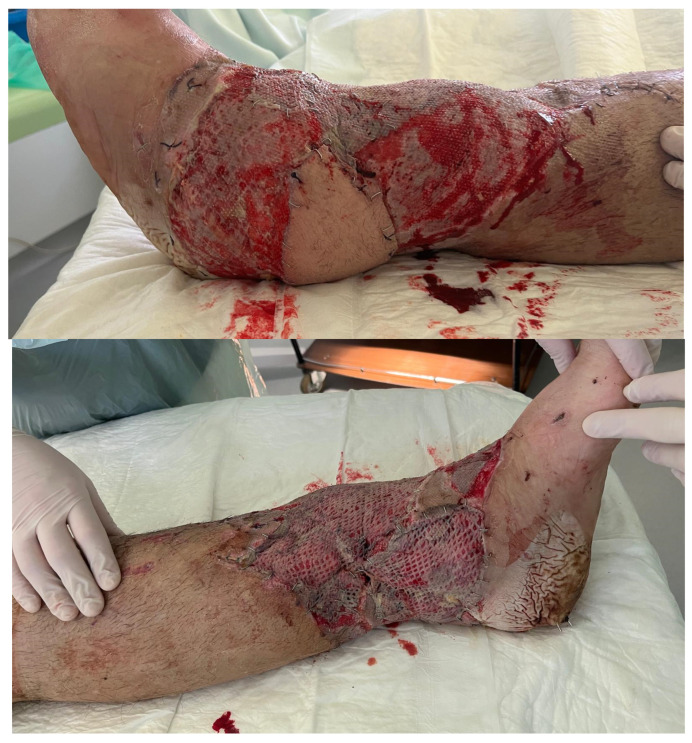

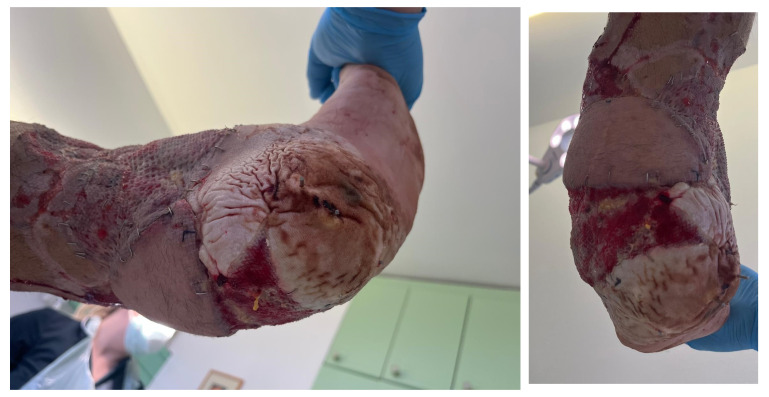
Medial, lateral and posterior part of the leg 10 days after reconstruction. Skin paddle random pattern propeller flap covering Achilles tendon.

**Figure 5:**
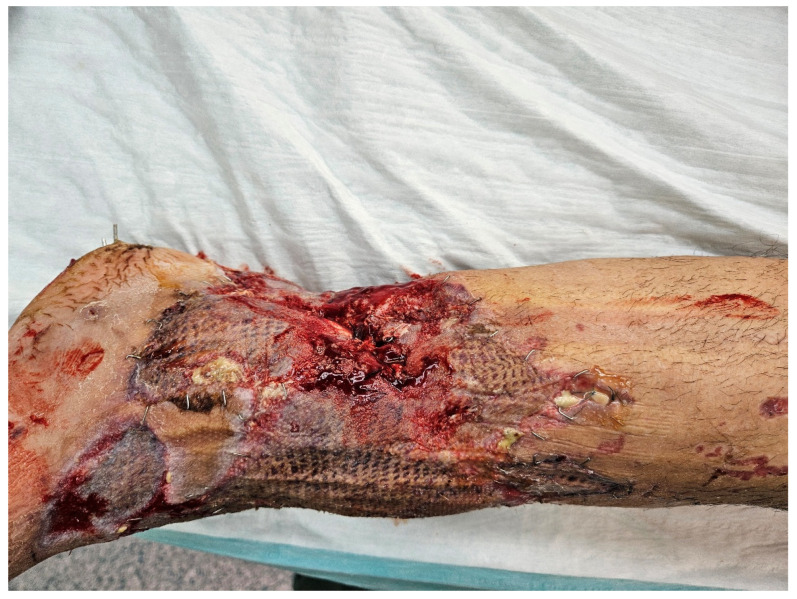
Medial aspect showing a postoperative complication with delayed healing requiring revision.

**Figure 6:**
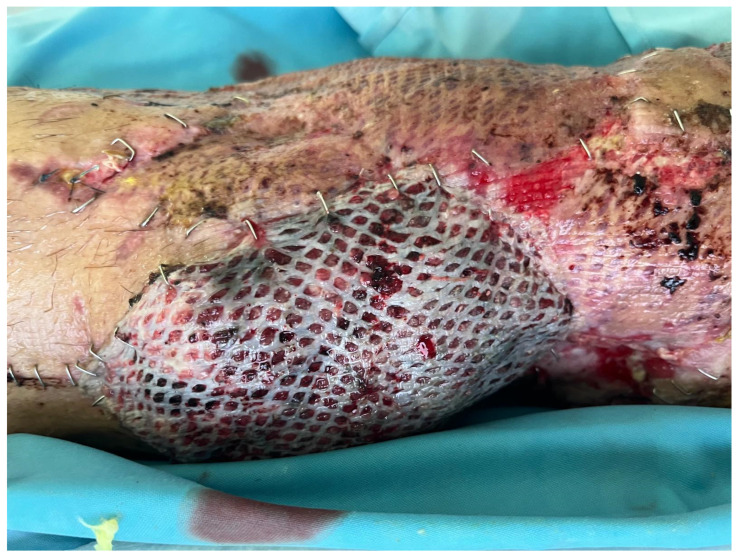
Early postoperative appearance a few days after reverse soleus flap reconstruction showing a viable flap and stable soft-tissue coverage of the medial defect.

**Figure 7:**
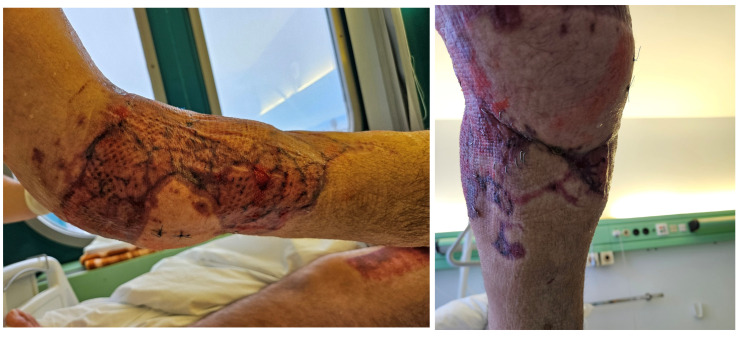
Clinical appearance three weeks after reconstruction showing complete flap viability, stable wound coverage, and satisfactory contour restoration of the distal lower leg.

**Figure 8:**
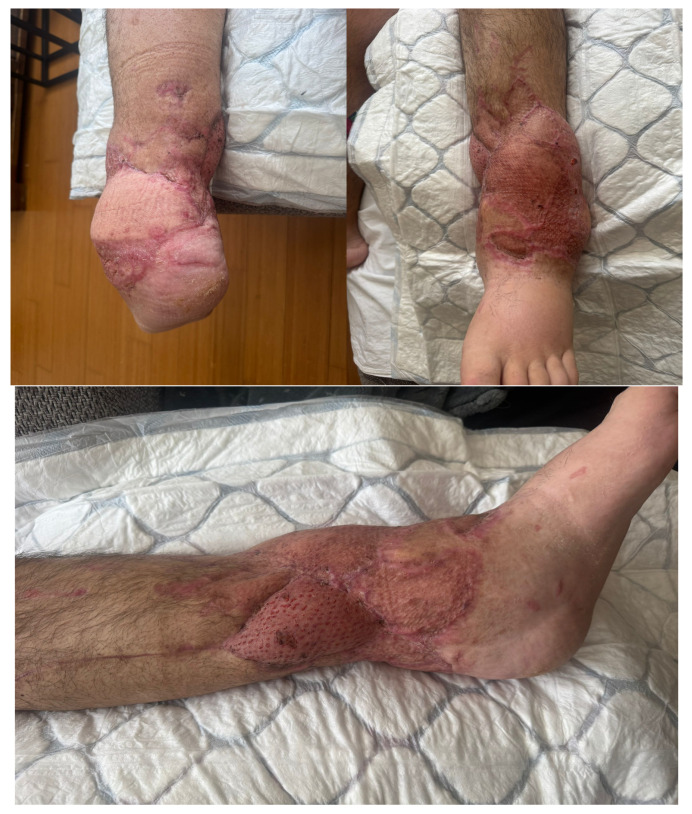
Clinical appearance 2 months after the last surgery.

The postoperative course was marked by a minor complication involving delayed healing along the medial proximal aspect of the reconstruction, most likely due to progressive deterioration of the previously injured tissue in that region, while the latissimus dorsi flap remained warm and well perfused on serial clinical assessment, with a consistently strong Doppler signal over the thoracodorsal pedicle throughout. Considering the local tissue condition and the presence of osteosynthetic material, prompt surgical revision was performed. Necrectomy of the devitalized area was followed by reconstruction using a reverse soleus flap, which achieved stable wound closure. Subsequent healing proceeded without further complications, with preserved flap viability, durable soft-tissue coverage, and successful limb preservation at last follow-up (Figure 4, Figure 5, Figure 6, Figure 7 and Figure 8). The plantar-flexed ankle position visible in postoperative photographs reflects intentional early postoperative immobilization to minimize tension on the soft-tissue envelope, not a fixed deformity. Progressive mobilization was initiated by postoperative day 5, and the patient was undergoing supervised rehabilitation with satisfactory wound maturation. Early physiotherapy focused on maintaining passive ankle and knee range of motion and isometric quadriceps exercises while strictly avoiding dependent dangling of the limb; thereafter, a graded dangling protocol and progressive partial weight bearing were introduced under supervision, with shoulder physiotherapy initiated once donor-site pain permitted to prevent stiffness and optimize function. The total hospital stay from admission to discharge was 64 days, including 7 days in the intensive care unit. At last follow-up, the patient reported being satisfied with limb function and the overall appearance of the reconstruction, with no limitations in activities of daily living and only mild subjective weakness during strenuous overhead activities. Shoulder examination on the donor side demonstrated preserved full range of motion.

## 3. Discussion

The design and elevation of a latissimus dorsi skin paddle without explicit preoperative perforator mapping represents a practical adaptation to the realities of acute high-energy trauma reconstruction, where contaminated, extensively burned, or severely traumatized tissue may make reliable imaging impossible. The decision to design the skin paddle empirically over the constant perforator zone was justified by adherence to Godina’s principle of early soft tissue coverage within 72 h to minimize infection risk and optimize flap incorporation, the anatomical certainty that the defined zone contains robust perforators, and the practical recognition that intraoperative Doppler failed to identify perforators in the contaminated skin, making a random-pattern approach within the anatomically predictable zone the most prudent option, a principle supported by translational studies demonstrating that latissimus dorsi skin paddles can be successfully designed according to random-pattern concepts without explicit perforator identification when standard anatomical guidelines are followed and by clinical series reporting reliable lower-extremity reconstruction using latissimus dorsi musculocutaneous flaps in acute high-energy trauma without routine preoperative perforator imaging [8,10,11]. We acknowledge that the intraoperative perfusion assessment in this case relied on clinical parameters that remain partly subjective (color, temperature, capillary refill, and bleeding characteristics), and future studies should incorporate quantitative perfusion assessment tools such as fluorescence-based angiography to more objectively characterize perfusion in random-pattern latissimus dorsi skin paddles. The postoperative medial necrosis was confined to previously traumatized native tissue at the flap–wound interface, while the latissimus dorsi flap and skin paddle remained fully viable on serial clinical assessment (color, temperature, capillary refill) and with a consistently strong Doppler signal over the thoracodorsal pedicle, findings that support progressive zone-of-injury deterioration rather than flap failure. Management with a local pedicled reverse soleus flap, rather than a second free tissue transfer, underscores the value of preserving local muscle options during primary reconstruction and anticipating staged revision in high-energy trauma. It is important to distinguish the present technique from conventional latissimus dorsi myocutaneous flap applications, where the skin paddle remains fixed on the muscle surface as a monitoring tool or composite unit. In our case, the skin paddle was secondarily mobilized through stepwise subcutaneous undermining and rotated as an independent local extension flap. In conventional lower-extremity reconstruction, the latissimus dorsi muscle and its skin paddle are typically transferred as a single composite unit that provides primary coverage and monitoring but cannot be independently reoriented to address a separate distal defect, so additional local or free flaps and/or skin grafting are frequently required for secondary wound components, particularly around the ankle and heel [1,11,12]. By contrast, in the present case the muscle component was used to cover the anterior tibial and hardware exposure while the skin paddle was later mobilized and rotated as a propeller-type extension to reconstruct the Achilles and posterior heel, extending the reach of a single free flap and obviating the need for a second free flap.

Recent cadaveric microvascular injection studies have established that the latissimus dorsi flap possesses highly consistent perforator locations within defined zones [4,5]. Specifically, Schaverien and colleagues demonstrated that within a region 9.5 to 15.4 cm from the posterior axillary fold and within 4.3 cm of the lateral border of the latissimus muscle, at least one musculocutaneous perforator ≥ 0.5 mm in diameter was present in 100% of specimens, establishing it as an anatomically constant perforator zone. Furthermore, in 58% of specimens, multiple perforators from the descending branch of the thoracodorsal artery were found within this zone, establishing it as a safe island for skin paddle design [4]. Watanabe and colleagues expanded this understanding by defining the three-dimensional angiosome architecture of the latissimus dorsi, demonstrating that the first vascular territory, supplied by the thoracodorsal artery and lateral intercostal perforators (9th and 10th intercostal), represents the primary perfusion zone and is directly connected to the dominant thoracodorsal pedicle, while the second territory, supplied by medial intercostal, subcostal, and lumbar perforators, receives blood flow from the first territory through choke vessels, enabling perfusion of skin extending down to the iliac crest. Importantly, the skin paddle can safely reach the iliac crest provided it includes the lateral 9th or 10th intercostal perforators that link the muscle and skin components of the first angiosome [5]. Although these findings are derived from cadaveric injection studies, their clinical relevance in the trauma setting is indirectly supported by consistently high success rates of latissimus dorsi musculocutaneous free flaps reported in clinical series of high-energy lower-extremity reconstruction, suggesting that the thoracodorsal perforator system may preserve sufficient perfusion even in challenging recipient conditions [11,13,14,15]. The skin paddle survived and integrated successfully in this case, validating the anatomical premise that random perfusion within the constant perforator zone is reliable.

A distinctive aspect of this reconstruction was the intraoperative decision to mobilize the skin paddle and rotate it 150 degrees to serve as a separate local extension flap covering the Achilles tendon and posterior heel defects. A 150° propeller-type rotation of the skin paddle was achieved without vascular compromise, consistent with reports that perforator-based propeller flaps can tolerate large arcs of rotation up to 180° when the pedicle is carefully dissected and perfusion is meticulously assessed, although higher complication rates have been noted in extremity flaps with arcs exceeding 150° [16,17]. This approach accomplished extended coverage without requiring either additional soft tissue transfer or reliance on split-thickness skin grafting alone. By using the skin paddle as a local extension, the need for a second muscle flap was eliminated, permitting preservation of the serratus anterior muscle despite the large overall defect size [18]. The limited latissimus dorsi harvest, combined with serratus sparing, permitted primary closure of the left dorsal donor site, as closure can be achieved primarily when the skin paddle width does not exceed approximately 10 cm and when the cranio-caudal extent respects the boundary of the 12th rib [1,5]. Conservative harvest and serratus sparing directly correlate with reduced seroma formation and reduced permanent shoulder dysfunction, with functional studies showing that preserved latissimus innervation maintains measurable strength and quality of life in the postoperative period [2,19].

The choice to maintain a skin paddle on the latissimus dorsi, rather than harvest muscle only and rely on skin grafting, is supported by robust evidence from the contemporary lower-extremity reconstruction literature [20,21]. Stranix and colleagues demonstrated that muscle flaps carrying a skin paddle had dramatically superior salvage rates following vascular compromise, with 35.7% of flaps with skin paddles successfully salvaged after take-back compared with only 4.5% of muscle-only flaps. This survival advantage reflects the crucial benefit of direct clinical monitoring, as the skin paddle permits visualization of color, turgor, and Doppler signals without reopening the flap, enabling earlier detection of vascular compromise and faster return to the operating room. Furthermore, regression analysis confirmed that the presence of a skin paddle did not increase overall flap complications or failure rates despite the additional metabolic demand, suggesting that the monitoring benefit outweighs any theoretical disadvantages [20]. While this study provides strong quantitative support that the presence of a skin paddle can improve muscle flap salvage after microvascular compromise in lower-extremity reconstruction, it is a single-center retrospective series demonstrating association rather than proof of causation, and its results cannot be directly extrapolated to guarantee salvage of an empirically designed random-pattern skin paddle in every trauma setting.

The latissimus dorsi flap has emerged as one of the most frequently used free flaps for extensive lower-extremity soft tissue defects in recent years, particularly for distal tibia, ankle, and heel reconstructions [13,14]. In a systematic analysis of 32 Gustilo Type IIIC injuries (defined by major arterial disruption and extensive soft tissue loss), free latissimus dorsi myocutaneous flaps were used in 40.6% of distal tibia fractures and achieved successful reconstruction in 75% of cases despite zero-vessel runoff. This rate of success is comparable to single-vessel Gustilo IIIB reconstructions, suggesting that latissimus dorsi flaps provide reliable salvage even in severely compromised vascular settings [13]. The latissimus dorsi is particularly suited for distal leg and ankle reconstruction because the flap provides large tissue volume suitable for obliterating extensive dead space created by comminuted fractures, the thoracodorsal pedicle is long and large-caliber permitting anastomosis well outside the zone of injury, the muscle atrophies over time providing better contour and footwear accommodation, the muscle is supremely hardy and survives in contaminated or chronically ischemic beds where fasciocutaneous alternatives might fail, and harvest is rapid enabling efficient two-team operative coordination in time-sensitive trauma [11,14,15].

The anterolateral thigh flap represents the most commonly used contemporary alternative for large lower-extremity defects, with advantages of thinness, low donor-site morbidity, and ease of supine positioning for simultaneous defect and flap preparation [14,15]. However, anterolateral thigh flaps have recognized limitations including highly variable perforator anatomy with 10–15% incidence of absent or anomalous perforators requiring intraoperative modification, technically tedious and time-consuming perforator dissection, relative thinness that may not optimally fill deep cavities in contaminated wounds, and dimensional limitations for very large defects unless bilateral chain-linked flaps are used [14]. A recent head-to-head comparison of extended latissimus dorsi versus bilateral chain-linked anterolateral thigh flaps for distal lower-extremity defects greater than 300 cm^2^ revealed that extended latissimus dorsi flaps had significantly shorter flap elevation, significantly shorter anastomosis time, and significantly shorter total operative time while achieving equivalent flap success rates [22,23]. Moreover, latissimus dorsi harvest does not require extensive intramuscular perforator dissection, making it more forgiving in the setting of variable anatomy or contaminated wounds [22].

The patient’s defect extended to the plantar surface and heel, regions requiring particular consideration in flap selection, as the plantar skin is uniquely adapted to high compressive forces requiring reconstruction that restores durable, wear-resistant coverage capable of withstanding shoe pressure and shear forces. A systematic review of 2684 plantar reconstructions revealed that muscle or musculocutaneous flaps comprised approximately 29% of free flap reconstructions, with latissimus dorsi being the most frequently reported muscle-based option. The latissimus dorsi was identified for plantar defects specifically because of its durability, high reliability in contaminated or infected settings, and ability to provide large volume without compromising coverage. Notably, latissimus dorsi flaps demonstrated low partial necrosis rates (0–8%) and zero complete flap loss in this literature review, indicating exceptional reliability in the mechanically demanding plantar environment [12]. While potential bulk remains a concern, several factors mitigate this: the latissimus dorsi muscle gradually atrophies reducing initial bulk, the split-thickness skin graft overlay provides a relatively thin final epidermis, staged debulking procedures can be performed if needed after 3–6 months when the flap has stabilized, and the advantages of durability and infection resistance in a contaminated wound with extensive bone exposure outweigh aesthetic considerations in the immediate postoperative phase [12].

Function-sparing approaches to latissimus dorsi harvesting have demonstrated excellent long-term outcomes in lower-extremity reconstruction. A consecutive 5-year single-surgeon series of 42 lower extremity reconstructions using split latissimus dorsi muscle flaps achieved 95% success across diverse defects including ankle and distal leg trauma, establishing their reliability for complex soft tissue resurfacing. The split latissimus dorsi technique preserves posterior thoracodorsal nerve innervation, yielding Medical Research Council grade-5 strength in all patients across three functional tests, eliminating traditional latissimus dorsi donor morbidity concerns during lower extremity salvage. Postoperative shoulder assessments revealed negligible impairment with superior functional outcome scores, confirming preserved latissimus function supports optional skin paddle inclusion for monitoring without functional penalty [24]. Similarly, the muscle-sparing descending branch latissimus dorsi free flap for lower extremity reconstruction respects the angiosome anatomy, maintaining innervation and preserving strength while still providing enough volume for defect coverage [25]. These studies support the principle that limiting muscle harvest and preserving innervation does not compromise flap viability or outcome while substantially reducing donor-site morbidity [19].

The principle of the skin paddle as a monitoring and functional backup is particularly important in the context of complex lower-extremity defects with multiplanar involvement. Economic analyses of free flap reconstruction in lower-limb trauma demonstrate that while acute hospitalization costs are substantial, long-term functional outcomes and quality-of-life measures favor limb salvage over amputation and prosthetic fitting, particularly when flaps include monitoring capability to detect and manage early vascular compromise [26,27]. Systematic reviews confirm that long-term outcomes of latissimus dorsi reconstruction combined with advanced fixation techniques such as the Ilizarov apparatus yield acceptable functional limb preservation with acceptable complication rates [27].

Early mobilization protocols after latissimus dorsi free flap reconstruction enhance flap vascularization and physiological conditioning. McGhee and colleagues’ systematic review of eight studies encompassing 197 lower-extremity free flap recipients demonstrated that early dangling (typically beginning by postoperative day 3) induces characteristic improvements in tissue oxygen saturation and hemoglobin content over 3–4 days, with 57% of flaps beginning dangling by postoperative day 3 without increased failure risk. These findings support the contemporary adoption of progressive weight-bearing protocols as safe and beneficial adjuncts to flap maturation in lower-extremity reconstruction [28].

Chimeric flap designs combining the latissimus dorsi with other components of the subscapular system represent advanced strategies for complex lower-extremity defects. The triple conjoined scapular-latissimus dorsi-groin flap has been successfully applied for massive lower-extremity degloving injuries, demonstrating the versatility of LD-based designs in extending coverage while maintaining vascular reliability [18]. Similarly, combined latissimus dorsi with artificial dermis and split-thickness skin graft has been used effectively for extensive degloving injuries in the lower extremity, providing robust bulk, vascularity, and flexibility for staged refinement [29]. These approaches underscore the principle that the latissimus dorsi can serve as the anchor component of complex, multi-element reconstructions tailored to address extensive multiplanar trauma.

## 4. Conclusions

This single-case report illustrates the potential feasibility of using a random-pattern latissimus dorsi skin paddle as an independently mobilizable local extension flap for extensive distal lower-extremity reconstruction. The technique leverages established anatomical knowledge of constant latissimus dorsi perforator zones to permit skin paddle design without mandatory preoperative perforator imaging, a practical advantage in contaminated or time-sensitive acute trauma reconstruction. The incorporation of a random-pattern skin paddle based on the constant perforator zone located 9.5 to 15.4 cm from the posterior axillary fold and within 4.3 cm of the lateral border, supported by cadaveric angiosome studies demonstrating 100% perforator presence in this zone, eliminates the need for routine preoperative imaging or intraoperative Doppler identification [10]. Limiting cranio-caudal muscle harvest to respect the 12th rib boundary adheres to three-dimensional angiosome architecture and avoids the poorly perfused third vascular territory while enabling primary dorsal donor-site closure. The innovative use of the latissimus dorsi skin paddle as a local extension flap, rotated to extend coverage to secondary defect zones such as the Achilles tendon and plantar heel, increases effective flap reach without requiring additional muscle sacrifice or supplementary flap transfer. The patient in this case achieved successful flap incorporation with complete survival and enabling functional limb preservation. The latissimus dorsi, with its large size, reliable blood supply, proven durability in contaminated wounds, and adaptability to custom flap designs, remains an exceptional workhorse option for extensive lower-extremity trauma reconstruction. Potential complications that merit systematic evaluation include partial skin paddle necrosis, venous congestion, and distal wound breakdown at the flap–wound interface, and particular caution is warranted in patients with severely compromised recipient vessels or an inadequate constant perforator zone, in whom this approach may be contraindicated. We acknowledge that the single-case nature of this report limits generalizability. Future prospective case series should aim to prospectively validate the random-pattern skin paddle approach within the constant perforator zone, formally evaluate the local extension flap principle for extending latissimus dorsi coverage in complex multiplanar defects, define specific indications, refine patient selection criteria, and evaluate long-term functional outcomes and patient-reported satisfaction using standardized functional, sensory, and aesthetic assessment tools, which may further establish these techniques as standard options in the reconstructive surgeon’s approach to lower-extremity injuries.

## Data Availability

The original contributions presented in this study are included in the article. Further inquiries can be directed to the corresponding author.
